# Relationship between e-cigarette media content and product use: A scoping review

**DOI:** 10.18332/tid/200547

**Published:** 2025-02-27

**Authors:** Shaikha Aldukhail

**Affiliations:** 1 Department of Preventive Dental Sciences, College of Dentistry, Princess Nourah bint Abdulrahman University, Riyadh, Saudi Arabia

**Keywords:** electronic cigarettes, e-cigarette, social media, advertising, promotional strategy

## Abstract

**INTRODUCTION:**

E-cigarettes have emerged as a popular alternative to traditional tobacco products, raising concerns about the potential public health impact of widespread exposure to e-cigarette content. This scoping review aimed to answer the question: ‘Is there an association between exposure to e-cigarette content on social or traditional media and product use among individuals?’.

**METHODS:**

The review was conducted in accordance with PRISMA-ScR guidelines. A literature search was performed in MEDLINE (PubMed), Cochrane, EMBASE, and ScienceDirect on 30 July 2023, including studies published from 2004 to July 2023. Only studies in English were included, focusing on participants exposure to e-cigarettes via media platforms. The review examined self-reported exposure to organic posts and promotions, with outcomes related to e-cigarette use (lifetime/ever and current/past 30-day use). The review also explored trends in media advertising and e-cigarette use during that period. The marketing platforms assessed included social media (Instagram, YouTube, Reddit, Facebook, Twitter, and Snapchat, websites), traditional media (television, movies, radio), and print media (magazines, newspapers).

**RESULTS:**

Of the 30 studies included in this review, 14 were longitudinal in design. The majority were conducted in the United States (n=27), with one study each from China, Germany, and Scotland. The prevalence of current and ever e-cigarette users varied across different regions and populations. The majority of studies covered in the review observed a significant association between e-cigarette marketing exposure and product use among various demographic groups. Multiple US national studies reported an upward trend in e-cigarettes use from 2011 to 2019. Longitudinal studies indicated a temporal relationship between e-cigarette marketing and subsequent product use, particularly among youth.

**CONCLUSIONS:**

This scoping review highlights the evolving landscape of e-cigarette media advertising and its potential correlation on product use. Exposure to e-cigarette content on traditional and social media was consistently associated with e-cigarette consumption among diverse populations.

## INTRODUCTION

Electronic cigarettes, commonly referred to as e-cigarettes or vaping devices, or ENDS (electronic nicotine delivery system), have rapidly emerged as popular tobacco products, particularly among adolescents. These battery-powered devices heat a liquid solution, commonly referred to as e-liquid or vape juice, which typically contains nicotine, propylene glycol, glycerin, and flavorings. When heated, the e-liquid is converted into an aerosol that users inhale^1^. E-cigarettes have now emerged as the most commonly used tobacco product among adolescents^2^.

Despite being marketed as a safer substitute for combustible cigarettes, the long-term health effects of vaping remain largely unknown^1^. Several studies have documented potential systemic and oral health consequences of vaping, including endothelial dysfunction, oxidative stress, increased blood pressure, dental caries, and periodontal inflammation^3-6^. E-cigarette vaping also poses risks such as nicotine-induced harm to the developing brain and exposure to toxic substances, including heavy metals, volatile organic compounds, and ultrafine particles^6,7^. Despite these risks, e-cigarettes have gained significant popularity, particularly among young people. According to the US Centers for Disease Control and Prevention (CDC), e-cigarette use among adolescents increased by 900% between 2011 and 2015 in the US^8^. Similar trends were reported by other countries as well^9-11^.

Major tobacco corporations predominantly own leading electric cigarette brands and employ the same marketing strategies as they do with their other tobacco goods to attract younger users. These tactics are designed to attract and engage vulnerable and younger audiences, potentially normalizing and glamorizing e-cigarette use among these demographics^12,13^. The use of diverse flavors, appealing product designs, and aggressive media campaigns – including the involvement of social media influencers and interactive content – are particularly effective in reaching and resonating with youthful demographics^14,15^.

Exposure to e-cigarette marketing on social and traditional media may significantly contribute to the increasing prevalence of their use. Social media platforms such as YouTube, Instagram, and Facebook have become key venues for e-cigarette marketing and promotion, often showcasing attractive imagery, celebrity endorsements, and enticing flavors^16,17^. Influencers and vaping enthusiasts further normalize and glamorize e-cigarette use by sharing photos, videos, and reviews of vaping products^18,19^. Traditional media outlets, including television, print, and radio, also play a role in exposing audiences to e-cigarette content^20,21^.

Although some countries have implemented regulations to restrict e-cigarette advertising^11,22,23^, these promotions continue to appear in various forms, such as event sponsorships and subtle product placements in pictures, films, and television shows, including those on platforms like Netflix and Instagram^24,25^. The tobacco industry is known for exploiting regulatory loopholes to market emerging products like e-cigarettes^26,27^.

The sharp increase in e-cigarette smoking over the past decade has raised growing concerns about the impact of e-cigarette marketing, particularly given the limited regulation of these products. The widespread exposure to e-cigarette content on social and traditional media has intensified worries about its potential public health impact, especially among youth, who may be more susceptible to marketing tactics and peer influence^28,29^.

Consequently, researchers, policymakers, and public health advocates are actively studying and debating the impact of media exposure on the increasing popularity of e-cigarettes and its implications for tobacco control efforts. The objectives of this scoping review are to explore the effects of social and traditional media content on e-cigarette usage and its growing popularity. The review will evaluate existing studies that investigate the relationship between media exposure and changes in e-cigarette use, to synthesize research findings and assess the scope of the current literature.

## METHODS

### Review design

This scoping review was structured using the Preferred Reporting Items for Systematic Reviews and Meta-Analyses extension for scoping reviews (PRISMA- ScR) guidelines^30^.

### Research question

A scoping review was performed to systematically map the existing research regarding the association between e-cigarette smoking and exposure via media platforms. The findings will be summarized, as presented in the results section, guided by the following research question:

‘Is there an association between exposure to e-cigarette content on social or traditional media and product use among individuals?’.

### Inclusion and exclusion criteria

Eligible studies had to be in English, original articles with accessible full text, and involve participants exposed to content about e-cigarettes through social or traditional media channels. Our PICO question used was: ‘In individuals aged 9 years or older, how does exposure to e-cigarette-related content on social and traditional media, compared to no exposure, affect e-cigarette use?’.

### Exposure to marketing platforms

This was assessed as reported social media (Websites, Instagram, YouTube, Reddit, Facebook, Twitter, and Snapchat) and/or traditional media, including broadcast (television, movies, or radio) and print (magazines or newspapers). Specifically, the required exposure and comparator involved self-reported instances of exposure to e-cigarette content on media platforms, encompassing both organic posts and promotional materials, such as advertisements or sponsorships. To ensure the study’s scope and relevance, we excluded studies that only assessed exposure to e-cigarette content from sources like point-of-sale locations, billboards, retail stores, events, tobacco brand pages, or retailer websites.

### The outcome of interest

This was e-cigarette use, which encompassed measures of :1) lifetime/ever, or 2) current use/past 30-day use. Additionally, studies were excluded if they did not provide information on susceptible age groups within the participant demographics or if they had insufficient outcome reporting.

### Search strategy

A literature search was performed in MEDLINE (PubMed), Cochrane EMBASE, and Science Direct. The search was conducted on 30 July 2023, and included studies from 2004 to July 2023. Search terms were adopted from the publications on e-cigarette use, media, and marketing. The e-cigarette-related search terms consisted of five keywords: ‘vape’, ‘electric cigarette,’ ‘vaping’, ‘e-cig’, and ‘e-cigarette’. The social and traditional media terms consisted of eight keywords: ‘social’, ‘YouTube’, ‘Instagram’, ‘broadcast’, ‘media’, ‘Twitter’, ‘Tik Tok’ and ‘Facebook’. The marketing search terms consisted of six keywords, including ‘advertising’, ‘promotion’, ‘marketing’, ‘influencer’, ‘intervention’, and ‘content’. The detailed search strategy can be found in Supplementary Table S1.

### Data extraction

The selected studies were coded in two phases. The first phase was title and abstract coding. In this phase, the studies were coded for their relevance to the topic of interest, which included e-cigarette-related social and traditional media posts and advertisements, and product use.

The full-text screening phase was conducted afterward, with the inclusion and exclusion criteria described in the previous section. Relevant variables were extracted, including citation details (authors, year of publication, and location), study design and methodology, population (age, sex, race, and sample size), marketing platforms assessed, exposure time frame, and e-cigarette use outcomes. The study design is reported as either longitudinal or cross-sectional. The study location is reported according to the country and state specified. The population or sample size is given as provided in the study’s analyses. Sex is given as male or female, while age is given in years or age group (i.e. 18–25 years).

The quality screening of the selected sources was conducted independently by two reviewers, with any conflicts at any stage of the screening process solved by revisiting the inclusion criteria and reaching a consensus. Due to the diverse nature and limited comparability of the studies, a scoping review was deemed the most appropriate method to offer a comprehensive overview of research on the relationship between media content and e-cigarette use among both youth and adults. This approach allowed to map differences in exposure and outcome measures.

## RESULTS

### Characteristics of studies

**Figure 1:**
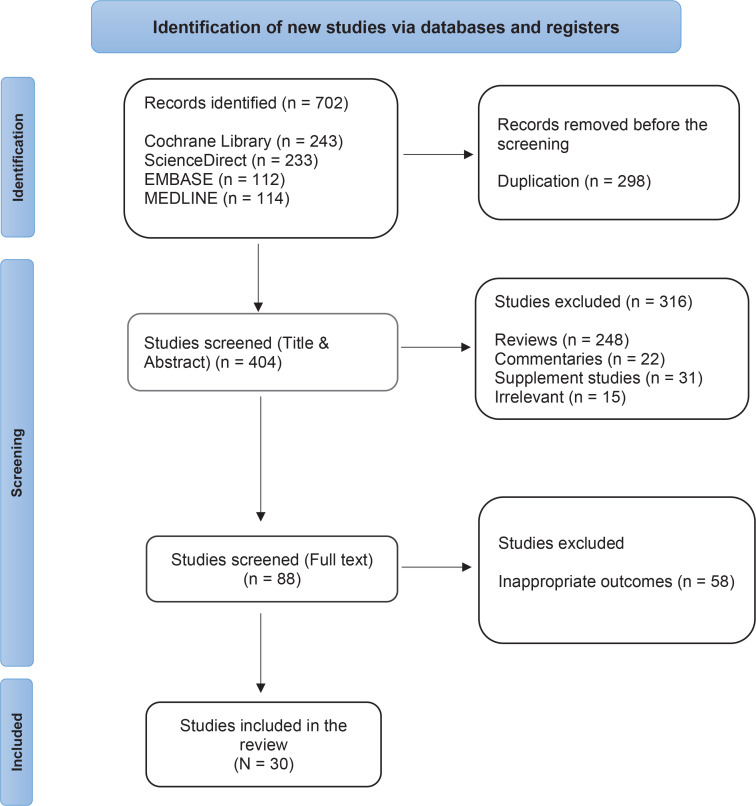
PRISMA- ScR flowchart diagram of study selection for the review of the association between e-cigarette use and exposure via media platforms

We initially identified 702 records from Cochrane (n=243), ScienceDirect (n=233), EMBASE (n=112), and MEDLINE (n=114). During the primary screening, we removed 298 duplicate records from the selected studies. After this step, we reviewed the titles and abstracts of the remaining studies, excluding 316 sources that did not meet the necessary criteria. These exclusions included reviews (n=248), commentaries (n=22), supplementary studies (n=31), and irrelevant studies (n=15). the exclusion criteria were based on the presence or absence of at least one of the topic keywords. In the next round of screening, we assessed the full texts of 88 studies for eligibility, resulting in the exclusion of 58 studies (Figure 1).

### Description of studies

Of the 30 original research articles included in this review, all were observational^31-60^. Fourteen of these studies employed a longitudinal design^31,32,34,35,37,39-43,45,51,53,60^, while the remaining studies were cross-sectional^33,36,38,44,46-50,52,54-59^. The majority, 27 studies, were from the US^31,32,34-41,43-55,^ ^57-60^. This review also included a single study from each of China^33^, Germany^42^, and Scotland^56^ (Table 1).

**Table 1:** Main characteristics of the studies included in the scoping review on the association between e-cigarette use and content exposure via media platforms (N=30)

*Authors Year Country*	*Study*	*Population*	*Intervention*	*Outcome*		
Sun et al.^31^ 2023 US	Longitudinal Data from the PATH Study, a cohort study of youth, and adults in the US. Waves used were 4, 4.5, and 5 (2017, 2018, 2019)	N=16671 12-14 years 40.7% 15-17 years 59.3% Female 48% NH-White 44.7% NH-Black 12.6% Hispanic 29.3%	Exposure to e-cigarette marketing in the past 30 days Social media or websites 2017 21.72% 2018 20.69% 2019 21.80% TV 2017 21.61% 2018 17.6% 2019 18.9% Newspapers or magazines 2017 14.7% 2018 11.4% 2019 10.0% Radio 2017 8.4% 2018 7.3% 2019 8.9%	E-cigarettes use	Past 12 months	Past 30-day
				Lifetime	2017 10.5%	2017 4.3%
				2017 14.9%	2018 13.2%	2018 6.8%
				2018 16.6%	2019 16.4%	2019 8.6%
				2019 19.6%		
				Findings - Exposure to e-cigarette content on social media or websites was significantly associated with past 12 months product use (AOR=1.7; p<0.005). Similarly with past 30-day use (AOR=1.5, p<0.05). - E-cigarette never smokers who were exposed to content via social media platforms were associated with subsequent ever e-cigarette use 12 months later (OR=2.1; p<0.001). - Exposure to e-cigarette advertisements on social media was significantly linked to ever e-cigarette use among never users (AOR=1.4, p<0.001).		
Wang et al.^32^ 2022 US	Longitudinal Data from the PATH Study, a cohort study of youth, and adults in the US. Waves used were 4, 4.5, and 5 from years (2017, 2018, 2019)	N=8548wave 4 (baseline) N=10073 wave 4.5 N=11641 wave 5 12-17 years Wave 4 Female 48.9% NH-White 52% NH-Black 13% Hispanic 24%	Exposure to e-cigarette marketing in the past 30 days Exposure was 60% 2017–2019 2017 TV 23.4% Online 21.3% Online exposure remained relatively stable from 2017 to 2019, TV exposure decreased during this period Print media 2017 15.2% 2019 9.1%	E-cigarettes use - Findings - Adolescents exposed to e-cigarette advertising were more likely to have ever used e-cigarettes than the not exposed: 9.6% vs 5.0% in 2018 and 13.1% vs 8.2% in 2019. - Similarly with current e-cigarette use, higher prevalence was reported among those exposed to advertising: 4.5% vs 2.1% in 2018, and 6.1% vs 3.2% in 2019 at follow-up. - Consistent associations were observed at follow-up for ever e-cigarette use (AOR=1.2; p<0.001) and current e-cigarette use (AOR=1.4, p<0.05).		
Dai et al.^33^ 2022 Shanghai China	Cross-sectional Data of students from 20 schools using probability sampling. (October -December 2019)	N=12470 13–18 years Chinese Female 47.6%	Exposure to e-cigarette marketing in the past 30 days Approximately 18.6% and 18.0% of the students were exposed to e-cigarette content through websites or social media platforms	E-cigarettes use Ever 1.1% Current 0.5% Findings - Exposure to e-cigarette content on social media was significantly associated with ever use (AOR=1.5; p<0.05), current use (AOR=1.6; p<0.05), compared to the not exposed.		
Do et al.^34^ 2022 US	Longitudinal Data collected in 2018 - baseline survey (n=3886) A follow-up study was conducted in 2019 (n=2304)	N=2304 13-20 years 63% 21-24 years 37% Female 75% NH-White 51.5% NH-Black 16% Hispanic 20%	Exposure to e-cigarette marketing At Baseline Any TV, retail, or online/social media ads or content 13-20 years 63.7% 21-24 years 58.3% TV advertising 13-20 years 24.3% 21-24 years 22.5% Online/social media advertising/content 13–20 years 42.9% 21-24 years 43.2%	E-cigarettes use Ever use 13-20 years 7.6% 21-24 years 12.8% E-cigarette user at follow-up 13-20 years 7.9% 21-24 years 11.1% Findings - The exposed via TV, retail, or online/social media at baseline were more likely to use e-cigarettes at follow-up (12.2%) than the not exposed (4.1%). - Youths with initial exposure to media content had over twice the odds of becoming recent e-cigarette smokers at follow-up (AOR=2.8; p<0.05) compared to those not exposed. - Youths exposed to TV (AOR=3.1; p<0.05) or online/social media (AOR=2.3; p<0.05) marketing at baseline showed higher odds of becoming recent e-cigarette smokers at follow-up, compared to the not exposed.		
Pokhrel et al.^35^ 2022 Hawaii US	Longitudinal The study used four waves of data from college students with 6-month intervals (2018–2020)	N=2335 18–25 years Female 55% NH-White 24% Pilipino 19% NHPI 20% Asian 26%	Exposure to e-cigarette marketing Higher initial levels of exposure to video, internet, or print marketing, as measured by cued-recall, were significantly associated with being female	E-cigarettes use - Findings Participants who experienced increased exposure to e-cigarette advertising were more likely to have higher rates of e-cigarette use (B=0.63, p<0.05).		
Struble et al.^36^ 2022 US	Cross-sectional Data of sexual minority and heterosexual young adults using a US national sample from the (PATH) Survey, Wave 3, (2015–2016)	N=14174 18–34 years Female 53% NH-White 71.5% NH-Black 9% Other 20%	Exposure to e-cigarette marketing Websites or social media Sexual minorities 18% Heterosexual 16% Newspapers or magazines Sexual minorities 16.3% Heterosexual 13.9% TV Sexual minorities 16% Heterosexual 16.1%	E-cigarettes use Current Sexual minority 8.8% vs heterosexual 5.4% Lifetime Sexual minority 57.6% vs heterosexual 44.9% Findings - Individuals recently exposed to content via websites or social media had higher odds of reporting current e-cigarette use (OR=1.3; p<0.05) compared to the not exposed. - Those exposed to marketing in newspapers or magazines (OR=0.7; p<0.05) or on television (OR=0.66; p<0.05) had lower odds of currently smoking e-cigarettes compared to those not exposed.		
Zheng et al.^37^ 2021 US	Longitudinal Data from the PATH Study, a cohort study of youth, and adults in the US Waves 2–4 (2014–2018)	N=6208 12-14 years 75% 15-17 years 25% Female 49% NH-White 52% NH-Black 14% Hispanic 23.5%	Exposure to e-cigarette marketing Online exposure 38.9% High social media use was correlated with higher odds of e-cigarette content exposure (OR=1.1; p<0.05)	E-cigarettes use Ever (wave 2) 6.70% Past 30 days (wave 4) 5.81% Findings -Model estimation suggested that previous social media use was significantly associated with higher e-cigarette use later (sequentially mediated by higher exposure to e-cigarette marketing and lower risk perception between the two-time points).		
Ali et al.^38^ 2020 US	Cross-sectional Data from the National Adult Tobacco Survey (NATS) linked to Kantar Media and National Consumer Study data (2013–2014)	N= 98746 18-24 years 9.6% 25-44 years 34.4% ≥45 years 56% Female 53% NH-White 71.5% NH-Black 8.6% Other 19.9%	Exposure to e-cigarette marketing TV 48% Magazines 86% From 2012 to 2014 TV ads increased from 0.4% to 1.8%, magazine ads increased from 0.8% to 4.1% Ages 25–44 years had the highest levels of marketing exposure	E-cigarettes use Ever 15.8% Current 2.6% Findings - TV exposure was correlated with greater use. - Every additional TV ad correlated with a 0.13% point increase in the likelihood of ever using and a 0.03% point increase in current vaping. - No relationship was reported between magazine ads and either ever or current e-cigarette use.		
Pokhrel et al.^39^ 2020 Hawaii US	Longitudinal Randomly selected college students were invited to participate (n=2622) 2 follow-up surveys were collected 6 and 12 months later (2017–2019)	N=2327 18-25 years Females 55% NH-White 24% Asian 26% Filipino 18% NHPI 21% Other 11%	Exposure to e-cigarette marketing Facebook 15% Instagram 24% Current vapers were more likely to encounter e-cigarette content across multiple social media platforms	E-cigarette use Current 30% Lifetime 63% Findings - Greater exposure to e-cigarette content on social media was associated with increased e-cigarette use at 6 and 12 months follow-up. - Baseline social media exposure indirectly affected e-cigarette initiation at 12 months (Indirect effect estimate=0.02; p=0.02). - A direct association was observed between social media and e-cigarette initiation at 12 months (Direct effect estimate=0.03; p<0.001). - A positive correlation was observed between social media use and lifetime e-cigarette use.		
Etim et al.^40^ 2020 California US	Longitudinal Used data from a tobacco marketing study A cohort of high school students from southern California recruited from two survey panels (2018 and followed up until 2019)	N=1060 15-20 years Female 49.7% Hispanic 75.2% Other 24.8%	Exposure to e-cigarette marketing TV or Internet There was no difference in baseline exposure to e-cigarette content	E-cigarette use Past 30 days (Females, 15 years) 0.17% Increased to 0.21% at 18 years, and then declined to 0.14% by age 20 years. Past 30 days (Males, 15 years) 0.21% Increased to 0.33% at 18 years, and then declined to 0.20% by age 20 years. Findings - No significant relationship was observed between exposure to e-cigarette content and current use among females. - A significant association was observed between exposure and use among males, particularly between ages 16 and 18 years. - At age 17 years, each additional unit increase in e-cigarette content exposure was associated with a 30% increase in the likelihood of vaping (p<0.05).		
Clendennen et al.^41^ 2020 Texas US	Longitudinal Data collected from two waves of the Marketing and Promotions across Colleges in Texas (M-PACT) project. (2014–2018)	N=3947 18-29 years Female 65% NH-White 35% NH-Black 8% Hispanic 31% Asian 19%	Exposure to e-cigarette marketing Facebook, Instagram, YouTube, Twitter, Snapchat, Reddit, and Pinterest	E-cigarette use Lifetime 57.4% Past 30-day 8.6% Findings - Exposure to marketing on social media was significantly linked to the ‘past 30-day use’ of e-cigarettes at the 12 months follow-up. - After controlling for other social media influences, exposure to any product marketing on Reddit significantly increased the likelihood of e-cigarette use (AOR=1.9; p<0.05).		
Hansen et al.^42^ 2020 Germany	Longitudinal A school-based survey conducted with a sample of German adolescents, with the baseline assessment occurring in 2016–2017 and a follow-up assessment conducted 12 months later	N=4529 16-19 years Female 49% Migrants 13.7%	Exposure to e-cigarette marketing TV and Internet Non-users 32% Users 51%	E-cigarettes use - Findings - Approximately 38% recalled seeing e-cigarette content at baseline, with 13.6% starting to vape within 12 months. - A significant correlation was identified between marketing exposure and later use (AOR=1.4; p=0.024). - Among those with high exposure to e-cigarette content, 20.5% initiated use during the study period. - In contrast, only 11.1% with no exposure initiated e-cigarette use.		
Kreitzberg et al.^43^ 2019 Texas US	Longitudinal Data collected from waves of the Marketing and Promotions across Colleges in Texas (M-PACT) project Students completed a baseline survey in 2014–2015 and three subsequent surveys 6 months apart	N=5478 18-29 years Female 51% NH-White 36.4% NH-Black 17% Hispanic 31.1%	Exposure to e-cigarette marketing TV, radio, Internet The average score for recent exposure fluctuated between 3.25 and 3.8	E-cigarette use Past 30-day: from 17.2% to 10.9% by follow-up Findings - Exposure to ENDS marketing in each previous wave consistently predicted subsequent use (β=0.07–0.10, p<0.001). - ENDS use in waves 2 and 3 was a predictor of reported marketing exposure in waves 3 and 4, respectively (β=0.07–0.09, p<0.001). - Although ENDS users were more likely to report marketing exposure than non-users, this reported exposure still predicted future vaping.		
Papaleontiou et al.^44^ 2019 US	Cross-sectional Data from the 2015 National Youth Tobacco Survey (NYTS)	N=17711 9-18+ years	Exposure to e-cigarette marketing TV, magazines and newspapers, Internet 38.7% via at least one channel TV 13.9% Internet 12.1% Magazines/ newspapers 8.8%	E-cigarettes use - Findings - Exposure to both traditional tobacco and e-cigarette marketing was significantly correlated with higher odds of current vaping (AOR=1.6; p<0.05).		
Camenga et al.^45^ 2018 Connecticut US	Longitudinal Data were drawn from 2 waves of school-based surveys of 3 high schools and 2 middle schools in Connecticut (2013–2014)	N=1742 Female 53.9% NH-White 88.1% NH-Black 3.1% Hispanic 4.9% Asian 5.8%	Exposure to e-cigarette marketing TV/radio 29.2% Magazines 19.4% YouTube 8.7% Facebook 7.6% Twitter 6.8% Other social media platforms 15.4%	E-cigarette use status By the 2nd wave, 9.6% of e-cigarette never users at 1st wave reported vaping Findings - Exposure to e-cigarette marketing on Facebook (OR=2.2, p<0.01) during wave 1 significantly increased the probability of vaping by wave 2.		
Simon et al.^46^ 2018 Connecticut US	Cross-sectional Students from 8 high schools in Connecticut, completed the survey in 2015	N=3473 12-14 years 40.7% 15-17 years 59.3% Female 51% NH-White 52.7% NH-Black 14.6% Hispanic 14.7%	Exposure to e-cigarette marketing Total advertising exposure, mean (SD): 2.1 (2.8) TV 32.7% Magazines 23.2% Social media 23% Radio 11.7% No exposure <1% of adolescents	E-cigarette use status Past 30-day 19% Findings - Higher levels of advertising exposure were significantly related to more frequent e-cigarette use.		
Ashford et al.^47^ 2018 Kentucky US	Cross-sectional Data collected by surveying women who reported using tobacco within the past 12 months. Quota sampling to achieve somewhat equal numbers of pregnant (n=101) and non-pregnant (n=99) participants (2014–2015)	N=200 18-45 years Female 100% NH-White 78% Other 22%	Exposure to e-cigarette marketing Media exposure 9.31% The most common sources of advertising exposure were television, social media, and internet blogs, with radio and internet news also being noted	E-cigarette use status Ever use 64.9% Findings - The logistic regression model assessing e-cigarette use showed overall significance (G^2^=24.6, p<0.001). - Key significant factors included age, race, and the level of media exposure to e-cigarette marketing. - Each 1-point increase in e-cigarette content exposure was associated with a 4% higher probability of ever use. Additionally, a 10-point increase in exposure corresponded to a 50% higher probability of being an ever vaper.		
Wagner et al.^48^ 2017 NJ US	Cross-sectional Data were collected from a 2015 survey of pregnant women, recruited via a national website survey service, Amazon Mechanical Turk (MTurk)	N=445 18-45 years Female (pregnant) NH-White 72.58% NH-Black 15.1% Other 12.4%	Exposure to e-cigarette marketing Internet, print, TV 83%	E-cigarette use status E-cigarette 6.52% Dual-use combustible cigarettes and e-cigarettes 8.54% Findings -74.6% of vapers reported switching to e-cigarettes after discovering their pregnancy. - Differences between usage groups were not significant regarding the likelihood of viewing advertisements.		
Pesko and Robarts^49^ 2017 US	Cross-sectional Data from the (NYTS) of students in grades 6 through 12 (2011–2014)	N=71012 11-17 years Female 50.1% NH-White 53% NH-Black 13% Hispanic 20%	Exposure to e-cigarette marketing Exposure via the Internet, newspapers/magazines, TV/movies, mean (SD) 1.91 (0.13)	E-cigarette use status Current 3.40% Findings - Current vaping among urban adolescents increased from 0.92% in 2011 to 8.62% in 2014 (p<0.001). In contrast, for rural adolescents it increased from 2.13% in 2011 to 4.26% in 2014 (p<0.05). - Urban adolescents experienced a 4-fold increase in current vaping from 2.4% to 8.6% from 2013 to 2014. - A 1-point increase in advertisement exposure was associated with 6.4 times higher odds of current vaping (p<0.001).		
Sawdey et al.^50^ 2017 Virginia US	Cross-sectional Data from a sample of college students was via a questionnaire in 2016	N=258 18-20 years 59% 21-24 years 33% 25+ years 8% Female 67% NH-White 49% NH-Black 21% Hispanic 12% Asian 16%	Exposure to e-cigarette marketing Facebook, Twitter, Instagram 43% peer posts 48.5% e-cigarette content	E-cigarette use status Lifetime 46% Current 16% Dual users (e-cigarettes and cigarettes) 7% Findings -Lifetime vaping was positively correlated with exposure to both peer posts (AOR=3.1; p<0.05) and social media marketing (AOR=3.0; p<0.05). - Current use was only significantly correlated with viewing peer posts on social media (AOR=7.6; p<0.05).		
Nicksic et al.^51^ 2017 Texas US	Longitudinal Prospective study, students participated in a youth tobacco surveillance study from 2014 to 2015 and completed a 6-month follow-up assessment	N=2488 12-17 years Female 49% Hispanic 54.5% NH-White 28% NH-Black 17.6%	Exposure to e-cigarette marketing TV 47.2% Radio or online radio 23.7% Internet 43.5%	E-cigarette use status Ever use (baseline) 18.5% Follow-up 3% new ever users Findings -The rate of current vaping declined from 5.8% to 3.5% at follow-up. - At baseline, content exposure via the Internet was significantly associated with both current use (AOR=2.2, p<0.05) and susceptibility to vape (AOR=1.72, p<0.05) at follow-up. - Students who recalled TV marketing were 60% more likely to ever vape compared to those who did not recall these ads (p<0.05).		
Pokhrel et al.^52^ 2017 Hawaii US	Cross-sectional A study of college students, a random selection of (n=1300) were invited to complete a screener survey. Those who met the eligibility criteria were invited to participate in the study (2016)	N= 470 18-25 years Females 65.2% NH-White 27.5% Asian 38.4% Filipino 16% Other 18%	Exposure to e-cigarette marketing Facebook ads 19% Instagram ads 16% E-cigarette-related posts Facebook 24% Instagram 20%	E-cigarette use status Current 24.5% Experimenter 33% Findings - E-cigarette marketing exposure on social media had an overall indirect effect on current vaping [estimate=0.05, p=0.008]. - Social media exposure to product marketing had a direct effect on current vaping, with greater exposure directly correlating to a higher likelihood of current vaping.		
Agaku et al.^53^ 2017 US	Longitudinal A nationally representative survey of adult non-users of cigarettes and e-cigarettes at baseline and 5-month follow-up (April–June and September– November 2014)	N=2191 18-24 years 11.3% 25-44 years 33.3% 45-64 years 35.5% 65+ years 19.8% Female 47.6% NH-White 69.4% NH-Black 10.3% Hispanic 7.1%	Exposure to e-cigarette marketing At baseline, adults viewed one of five popular e-cigarette ads via video Among those who did not smoke cigarettes or e-cigarettes at baseline, 16.6% reported exposure, mean score of 2.77	E-cigarette use status Among non-users at baseline who were exposed to these ads, the incidence rate of e-cigarette use at follow-up was 2.7% Findings - Adults who did not smoke cigarettes or e-cigarettes initially, the receptivity to e-cigarette marketing was significantly correlated with vaping at follow-up (AOR=1.6, p<0.05). - Males had lower odds of starting e-cigarette use at follow-up compared to females (AOR=0.4; p<0.05). - The attributable risk percentage of such marketing on vaping initiation was 59%.		
Singh et al.^54^ 2016 US	Cross-sectional Data analyzed from (NYTS), a survey of students in grades 6 through 12 (2014)	N=22007 9-18 years Female 49.5%	Exposure to e-cigarette marketing Middle school students current e-cigarette users’ exposure via the Internet 27.3% sometimes, 31.4% mostly or always High school students 38.1% sometimes, 18.7% mostly or always Similar patterns of exposure were observed across other sources of e-cigarette advertising (newspapers/magazines, TV/movies)	E-cigarettes use - Findings - Middle school students ‘sometimes’ exposed to e-cigarette content on the Internet had high odds of current use (AOR=1.4; p<0.05), and even higher odds with those exposed ‘mostly/always’ (AOR=2.9; p<0.05). -Frequent exposure to marketing in newspapers/magazines (AOR=1.7; p<0.05) and TV/movies (AOR=1.8; p<0.05) was associated with higher odds of current vaping. - High school students had over twice the odds of currently vaping when frequently exposed (AOR=2.02; p<0.05). - Occasional exposure to ads in newspapers/magazines was linked to 26% higher odds of current vaping (p<0.05), and frequent exposure also increased these vaping odds (71%; p<0.05). - High school students experienced 54% higher odds of current vaping with frequent exposure to e-cigarette content on TV/movies (p<0.05).		
Dai and Hao^55^ 2016 US	Cross-sectional Data analyzed from (NYTS), a survey of students in grades 6 through 12 (2014)	N=2149 9-18+ years Female 49.5% NH-White 45.5% NH-Black 15.5% Hispanic 27.5%	Exposure to e-cigarette marketing Internet 38.6% Newspapers/magazines 29.6% TV/movies 35.4% Among adolescents with moderate to high exposure, 32.3% exposed via one channel, 24.8% two channels, 20.3% three channels, 22.6% four channels	E-cigarette use status Current 9.4% Former 10.4% Findings - The prevalence and frequency of vaping increased with elevated exposure to product marketing, i.e. increased exposure to Internet content had a vaping rate of 17%, compared to 6.7% with low exposure. - Frequent vaping was more common among those who saw Internet e-cigarette content often, (3.8%) with high exposure. Similarly with exposure via newspapers/magazines and TV/movies. - Current vaping was correlated with high exposure via the Internet (OR=3.1), newspapers/magazines (OR=2.5), and TV/movies (OR=2.1) compared to low exposure (p<0.0001). - Higher exposure to Internet marketing (AOR=1.9) continued to observe a significant correlation with increased product use, whereas exposure through newspapers and TV/movies was not as significant.		
Best et al.^56^ 2016 Scotland	Cross-sectional Data collected as part of a 6-year multi-modal study. Employing a school-based survey with schools selected to represent two levels of urbanization and two tiers of social deprivation (high versus medium/low) (2015)	N=3808 10.83-18.67 years Female 49.7% White 92.3%	Exposure to e-cigarette marketing Internet 68.5 % Other channels (TV, radio, newspapers, magazines) 45.5 %	E-cigarette use status Ever 18.8% Findings - In the unadjusted model, all forms of content recall were significantly associated with the likelihood of e-cigarette use. - Internet content exposure had over twice the odds (OR=2.02; p<0.05) of e-cigarettes ever use, compared to no recall. - After adjusting for covariates, the model did not observe a significant correlation between content recall (via print media, TV, or billboards) and previous product use.		
Mantey et al.^57^ 2016 US	Cross-sectional Data analyzed from (NYTS), a survey of students in grades 6 through 12 (2014)	N=21491 9-18+ years Female 49.8% NH-White 53.2% NH-Black 14.6% Hispanic 21.95%	Exposure to e-cigarette marketing Internet 39.8% TV/movies 36.5% Print 30.4%	E-cigarette use status Current 9.3% Ever 19.8% Findings - Exposure to content through the internet was significantly correlated to ever vaping (AOR=1.6; p<0.05), as was print (AOR=1.2; p<0.05), and TV/movies (AOR=1.2; p<0.05). - Similarly, current vaping was 68% more likely among those exposed via the internet (p<0.05), 36% via print media (p<0.05), and 41% via TV/movies (p<0.05).		
Emery et al.^58^ 2014 US	Cross-sectional The data for this study were collected through a survey created by the Health Media Collaboratory at the University of Illinois, Chicago. The survey, administered by the GfK Group and involved a representative sample of US adults (2013)	N=17522 18+ years Females 52% NH-White 68.1% NH-Black 11.5% Hispanic 13.5% Other 6.9%	Exposure to e-cigarette marketing Television, radio, print media, and online social media 47% Television 66% Radio 19% Email 13% Internet search engines 11% Facebook 9%	E-cigarette use status Current 5.10% Ever 14.8% Findings - E-cigarette users were 337% more likely to encounter e-cigarette information through TV viewing platforms, 53% on radio, 386% on YouTube, 1697% on Twitter, 364% on Facebook, and 941% on Tumblr (p<0.05). - Tobacco users, younger individuals, males, those with higher education degrees, and those who frequently use social media were more likely to get passive exposure to e-cigarette marketing.		
Kim et al.^59^ 2013 Florida US	Cross-sectional A survey targeting adult smokers and recent quitters (within the past 12 months) in Florida Participants were recruited from comScore’s internet panel (2013)	N=519 18-24 years 13.9% 25-39 years 34% 40-64 years 49.4% 65+ years 2.7% Female 45% NH-White 64.2% NH-Black 12.7% Hispanic 18.1%	Exposure to e-cigarette marketing Television ads 60%	E-cigarette use status Used ENDS the past year 34.4% Current 23.2% Findings - Ever ENDS users reported significantly higher exposure to advertisements (70.8%) compared to non-users (58.3%). - Ever ENDS users also demonstrated notably greater receptivity to the Blu e-cigarette ads.		
Baumann et al.^60^ 2013 Alabama US	Longitudinal The study examined data from adult cigarette smokers who were hospitalized, with participants recruited in monthly cohorts over a nine-month period. Recruitment was based on the daily patient census at the hospital (December 2012 - September 2013)	N=979 19-80 years Female 47% NH-White 55.5% NH-Black 41.9%	Exposure to e-cigarette marketing Internet NH-White 13% NH-Black 6% Radio/TV NH-White 73% NH-Black 67%	E-cigarette use status NH-White 64% NH-Black 30% Findings - NH-White participants had over 5 times greater odds of using e-cigarettes compared to NH-Blacks (p<0.0001). - A significant interaction between race and advertisement exposure in predicting e-cigarette use remained. Specifically with vaping among NH-Blacks (p=0.006). - Increased exposure of (10 advertisements) was linked to a 6% rise in the likelihood of vaping.		

NH-White: Non-Hispanic White. NH-Black: Non-Hispanic Black. AOR: adjusted odds ratio. ENDS: Electronic Nicotine Delivery Systems.

Many US national studies utilized data from the National Youth Tobacco Survey (NYTS)^44,49,54,55,57^ or the Population Assessment of Tobacco and Health (PATH) surveys^31,32,36,37^.

Studies included investigated diverse populations. The most analyzed age group is adolescents in middle and/or high school (16 studies)^31-34,37,40,42,44-46,49,51,54-57^, followed by young adults (i.e. college students) (7 studies)^34,35,39,41,43,50,52^. Two studies analyzed the marketing and e-cigarette use among pregnant women^47,48^. All the included studies were journal articles published from 2013 to 2023. Two studies were published in 2013^59,60^, one in 2014^58^, four in 2016^54-57^, six in 2017^48-53^, three in 2018^45-47^, two in 2019^43,44^, five in 2020^38-42^, one in 2021^37^, five in 2022^32-36^, and one in 2023^31^ (Table 1).

### Trends in e-cigarette use

Multiple US national studies reported an upward trend in e-cigarette use from 2011 to 2019^31,32,34,38,49,57,58^. In 2013, approximately 5% of US adults were currently using e-cigarettes, while 15% had used them at some point^58^. In 2014, 9% of US youth were current e-cigarette users, and 20% had ever used them^57^. In the US, urban youth’s current e-cigarette usage rose sharply, from 0.92% to 8.62%, and among rural youth, it increased from 2.13% to 4.26%, from 2011 to 2014. Notably, the prevalence of current e-cigarette smoking among these youths quadrupled from 2.42% in 2013 to 8.62% in 2014^49^. A national study reported that in 2017, 15% of US youth were lifetime e-cigarette users, rising to 16.6% in 2018 and 19.6% in 2019. While past 30-day usage rates were 4.3% in 2017, 6.8% in 2018, and 8.6% in 2019^31^ (Table 1).

Among pregnant women in the US, e-cigarette use was 6.52% in 2015, with about 75% of these women switching to e-cigarettes upon learning of their pregnancy^48^. In Texas, 57.4% of college students reported lifetime e-cigarette use in 2018^41^. Between 2017 and 2019, college students in Hawaii reported a 30% rate of current e-cigarette use and a 63% rate of lifetime use^39^ (Table 1).

Moreover, in Scotland, approximately 19% of youth reported having tried e-cigarettes in 2015^56^. Finally, in China, 1.06% of youth were ever users of e-cigarettes, and 0.50% were current users as of 2019^33^ (Table 1).

### Association between exposure to e-cigarette marketing on social or traditional media and product use

A US national study reported that adult e-cigarette users in 2013 were more likely to encounter e-cigarette information through TV (OR= 3.4; p<0.05), radio (OR=1.5; p<0.05), YouTube (OR=3.9; p<0.05), Twitter (OR=17; p<0.05), and Facebook (OR=3.6; p<0.05) compared to non-users^58^. Another US national study found similar associations in 2014; youth who reported baseline exposure to e-cigarette content on TV (AOR=3.1; p<0.05), or online (i.e. social media) (AOR=2.3; p<0.05) were more likely to become a ‘past 30-day’ e-cigarette smoker later at follow-up, compared to the not exposed^55^ (Table 1). Further, it was found that between 2011 and 2014, an increase in exposure level (e.g. from ‘rarely see advertisements’ to ‘sometimes’, or from ‘sometimes’ to ‘most of the time’) was correlated with 6.4 the odds of current e-cigarette usage among adolescents in the US^49^. Similarly, a 2016 study found that e-cigarette content exposure through the internet (AOR=1.6; p<0.05), print media (AOR=1.2; p<0.05), and TV/movies (AOR=1.2; p<0.05) was significantly associated with ever using e-cigarettes. These trends were also observed for current e-cigarette use^57^. Between 2017 and 2019, a national US study reported that ‘past 12-months’ and ‘past 30-days’ e-cigarette use were significantly associated with exposure to e-cigarette marketing on social media/websites (AOR=1.6; p<0.05) and (AOR=1.5; p<0.05), respectively^31^ (Table 1).

A study of Virginia college students found that in 2016 ‘lifetime e-cigarette use’ was associated with exposure to product content through peer posts (AOR=3.1; p<0.05) and social media content (AOR=3.0; p<0.05), compared to non-exposure. While current product use was found to be significantly associated with exposure to social media peer posts^50^. Among pregnant women in Kentucky from 2014 to 2015, each one-point increase in exposure to e-cigarette advertisements was associated with a 4% increase in the likelihood of being an ever user. For every 10-level increase in exposure, these women had a 50% higher likelihood of being ever e-cigarette smokers^47^. During the same period (2014–2015) in Texas, college students who were exposed to ENDS content in a previous wave were more likely to use products in the following wave^43^ (Table 1).

In Germany (2016–2017), advertisement viewing was significantly correlated with subsequent e-cigarette use (AOR=1.4; p<0.05). Additionally, 20.5% of adolescents who reported high exposure levels (exposure level = 4) used e-cigarettes for the first time during the study period^42^. Likewise, in China in 2019, adolescents’ electric cigarette content viewing on social media was significantly correlated with lifetime vaping (AOR=1.5; p<0.05) and current vaping (AOR=1.6; p<0.05), compared to those who were not exposed (Table 1).

### Trends of media advertising for e-cigarettes in the US

US national studies covered in this review show that during the past decade, e-cigarette advertising shifted from traditional media (TV, radio, magazines, and newspapers) to the internet and social media. During 2012–2014, 48% and 86% of US adults were exposed to e-cigarette advertisements on TV and in magazines, respectively^38^. However, another report found that by 2019, only about 19% were exposed to e-cigarette advertisements on television and 10% in newspapers or magazines in the US^31^. Exposure from print media in the US decreased from 15.2% in 2017 to 9.1% in 2019^32^ (Table 1).

In 2015, about 14% of adolescents in the US reported internet exposure to e-cigarette advertisements^44^. While another study found that social media exposure among US adolescents was reported to be 22% in 2019^31^. Another study reported that between 2014 and 2018, increased social media use increased the odds of adolescents’ e-cigarette advertisement viewing on social media or websites (OR=1.1; p<0.05)^37^ (Table 1).

## DISCUSSION

This scoping review aimed to map the available empirical literature on the correlation between e-cigarette advertising and product use. The majority of the studies observed a significant association between e-cigarette content encounters on social or traditional media and product use among various demographic groups. The findings offer valuable insights into trends in e-cigarette media advertising over the past decade and the corresponding patterns of e-cigarette use across different populations.

Regarding e-cigarette use trends, the prevalence of ‘current and ever e-cigarette’ users varied across different regions and populations. The US exhibited a substantial increase in e-cigarette use, both among adults and adolescents, from 2013 to 2019^31,32,34,38,49,57,58^. Disparities existed among demographic subgroups in both e-cigarette use and product-related media exposure^34-36^. Higher vaping prevalence was also reported among cigarette smokers^60^. Notably, the prevalence of e-cigarette use among pregnant women in the US was concerning, particularly since 3 out of 4 cigarette smokers switched to e-cigarettes after learning about their pregnancy, raising potential public health implications^48^. A possible explanation for this behavior is the belief that e-cigarettes are a safer alternative to cigarettes and may assist in smoking cessation^61^. On the other hand, Chinese adolescents reported lower e-cigarette use compared to their German, Scottish, and US peers^31,33,42,56^. This discrepancy could be due to cultural differences, or that students may not be freely reporting their smoking habits for fear of violating school anti-smoking regulations^33^.

The association between exposure to e-cigarette content on social or traditional media and e-cigarette use, was consistently observed in almost all studies included in the review^31-47,49,51-57,59,60^. Adults and adolescents exposed to e-cigarette advertising on multiple media channels, such as TV, radio, and social media, showed higher odds of using e-cigarettes compared to non-exposed^31-34,42,45,54,55,57^. These findings underscore the influential role of media in shaping individuals’ attitudes and behaviors toward e-cigarette consumption. Furthermore, longitudinal studies suggest a temporal relationship between exposure to e-cigarette marketing and subsequent e-cigarette use, particularly among youths^31,42,43,45,53^. One national study reported that the population-wide attributable risk percentage from exposure to e-cigarette advertisements was 22.6% and the attributable risk percentage for e-cigarette initiation from exposure to e-cigarette advertisements was about 59% in the US^53^. Exposure to e-cigarette advertising in the ‘past 30 days’ was associated with subsequent lifetime e-cigarette use a year later among adolescents who had never tried e-cigarettes before in the US^31^. This association highlights the potential impact of advertising on the initiation and sustained use of e-cigarettes among vulnerable populations.

The observed shift in advertising media assessed in the studies included in the review highlights the growing influence of digital platforms in the marketing of e-cigarettes. In the early years of the review period, traditional media, such as TV, radio, magazines, and newspapers, were the primary channels for e-cigarette advertising^57-60^. However, by the end of the decade, the internet and social media emerged as prominent advertising channels for tobacco products and emerging products like e-cigarettes, reaching a wide and diverse audience^31,62^. This may be due to the tobacco industry’s tendency to exploit regulatory loopholes and reallocate marketing funds to less regulated channels^63^.

The review underscored that exposure rates to e-cigarette ads on TV and in magazines were high during 2012–2014, but these rates declined in more recent years, particularly in the US, from 2017 to 2019^32,38,57,58^. Conversely, internet and social media advertising witnessed a steady increase in exposure rates over time. About, 43% of US youths were exposed to social media ads in 2018^34^, compared to 12% in 2015^44^. These findings align with the increasing popularity and accessibility of internet-based platforms, especially among young adults and adolescents. The popularity of different social media platforms has also changed over the past decade. One of the most explored social media platforms in this review was Facebook^39,41,45,50,52,58^. In 2013, Facebook was the dominant social media platform among youth. However, its popularity has since declined, and it is now less popular than TikTok, Snapchat, YouTube, and Instagram^62^. This shift reflects the dynamic nature of advertising strategies and the industry’s adaptability to evolving media landscapes^14,16,63^. Exposure to social media e-cigarette content varied among individuals. Tobacco users, young individuals, men, higher SES, frequent users of social media, and those who spend more time online are more likely to have passively been exposed to e-cigarette content^46,58^.

### Limitations

Limitations of this study include variations in data collection methods and study designs, which may have influenced the comparability of findings. The majority of studies included in the review were from the US, with only a few from other countries. This limited geographical representation may restrict the generalizability of the findings to more diverse populations. There is also a potential risk of publication bias, as studies with significant results are more likely to be published, possibly leading to an overestimation of the association between media exposure and e-cigarette use. Additionally, recall bias could be a concern for self-reported data, as participants are asked to remember past behaviors or exposures. While the study mentions independent quality screening of selected sources by two reviewers, specific quality assessment methods were not detailed in this review. Unlike a systematic review, we included all relevant articles without attempting to synthesize evidence based on methodological quality from the outset. Furthermore, the rapidly evolving nature of media landscapes presents challenges in accurately capturing all forms of advertising.

### Future research

Future research on the associations between e-cigarette advertising and product use should adopt a more holistic approach by considering a wider range of influencing factors, such as the role of different media platforms and the effects on various nations and demographic subgroups. Expanding the geographical scope to include more diverse populations would also improve the generalizability of the findings. Additionally, a future systematic review and meta-analysis of the existing literature would be valuable for synthesizing evidence, identifying consistent patterns, and drawing more robust conclusions about the influence of e-cigarette advertising on usage across different contexts and populations.

## CONCLUSIONS

This scoping review sheds light on the changing landscape of e-cigarette media advertising and its potential correlation to e-cigarette use. Exposure to e-cigarette content on traditional and social media was consistently associated with e-cigarette use among diverse populations. The evidence presented here emphasizes the need for continued research, robust regulatory measures, and targeted public health campaigns to address the growing public health concerns associated with e-cigarette use and its marketing practices. Future research should explore deeper into the specific content and strategies employed in digital media platforms, as well as the effectiveness of regulations aimed at curbing the influence of e-cigarette advertising on vulnerable populations, particularly youths.

## Supplementary Material



## Data Availability

The data supporting this research can be found in the Supplementary file.
