# Supplementary material for: Relationship between e-cigarette media content and product use: A scoping review
Source: Tob Induc Dis. 2025 Feb 27;23:10.18332/tid/200547. doi: 10.18332/tid/200547 (PMC11866807; doi:10.18332/tid/200547)
Supplement: Supplementary file 1 [file TID-23-20-s1.pdf]

Supplementary Table S1: Search string for each database:

	Concept/Terms	Keywords
1) **MEDLINE (pubmed)**	E-cigarette<br><br>Social and traditional media<br><br>Marketing	("vape" OR "electric cigarette" OR "vaping" OR "e-cig" OR "e-cigarette")<br>AND<br>("social" OR "YouTube" OR "Instagram" OR "broadcast" OR "media" OR "Twitter" OR "Tiktok" OR "Facebook")<br>AND<br>("advertising" OR "promotion" OR "marketing" OR "influencer" OR "intervention" OR "content")
2) **Cochrane**	E-cigarette<br><br>Social and traditional media<br><br>Marketing	(vape OR "electric cigarette" OR vaping OR "e-cig" OR "e-cigarette")<br>AND<br>(social OR YouTube OR Instagram OR broadcast OR media OR Twitter OR Tiktok OR Facebook)<br>AND<br>(advertising OR promotion OR marketing OR influencer OR intervention OR content)
3) **EMBASE**	E-cigarette<br><br>Social and traditional media<br><br>Marketing	('vape' OR 'electric cigarette' OR 'vaping' OR 'e-cig' OR 'e-cigarette')<br>AND<br>('social' OR 'YouTube' OR 'Instagram' OR 'broadcast' OR 'media' OR 'Twitter' OR 'Tiktok' OR 'Facebook')<br>AND ('advertising' OR 'promotion' OR 'marketing' OR 'influencer' OR 'intervention' OR 'content')

Supplementary Table S1: Search string for each database:

**4) Science Direct**	E-cigarette<br><br><br><br>Social and traditional media<br><br><br>Marketing	("vape" OR "electric cigarette" OR "vaping" OR "e-cig" OR "e-cigarette")<br>AND<br>("social" OR "YouTube" OR "Instagram" OR "broadcast" OR "media" OR "Twitter" OR "Tiktok" OR "Facebook")<br>AND<br>("advertising" OR "promotion" OR "marketing" OR "influencer" OR "intervention" OR "content")

© 2025 Aldukhail S.
